# Piezoelectric Nanocoatings on Bio-Interfaces: Microenvironment Remodeling, Biofilm Disruption, and Immunomodulatory Integration

**DOI:** 10.3390/microorganisms14081822

**Published:** 2026-08-18

**Authors:** Yuemeng Li, Lixin Tang, Pengfei Gao, Jinhang Li, Xiaolin Sun, Jiao Fang

**Affiliations:** Department of Oral Implantology, Hospital of Stomatology, Jilin Provincial Key Laboratory of Sciences and Technology for Stomatology Nanoengineering, Jilin University, Changchun 130021, China; yuemeng25@mails.jlu.edu.cn (Y.L.); tanglx25@mail.jlu.edu.cn (L.T.); gaopf24@mails.jlu.edu.cn (P.G.); lijh3321@mails.jlu.edu.cn (J.L.)

**Keywords:** piezoelectric nanocoatings, Infectious microenvironment, biofilm, antibiotic-independent eradication, surface engineering

## Abstract

Implant-associated infections (IAIs) persist as a critical complication in biomaterial transplantation, driven by rapid microbial colonization, biofilm encapsulation, and escalating antibiotic resistance. Conventional antibiotic-based treatments and passive antimicrobial coatings often fail to achieve long-term infection control due to limited biofilm penetration, localized cytotoxicity, and active agent depletion. To overcome these limitations, piezoelectric nanocomposite coatings utilize a dynamic, stimulus-responsive framework that converts physiological mechanical forces or external ultrasound into localized electrical signals. These surface-bound electric fields systematically mitigate bacterial adhesion, eradicate mature biofilms via targeted reactive oxygen species (ROS) generation, disrupt microbial metabolic pathways, and favorably modulate the peri-implant immune microenvironment while supporting host tissue repair. This review evaluates the material design principles and classifications of inorganic, organic, and hybrid piezoelectric nanocoatings. We detail their multifaceted antibacterial mechanisms and trace their therapeutic potential in orthopedic and dental implants, as well as wound management. Lastly, we analyze current engineering bottlenecks to chart a clear trajectory for their clinical translation.

## 1. Introduction

Implant-associated infections (IAIs) represent a critical failure mode in modern biomaterial transplantation, primarily driven by the rapid surface colonization of opportunistic pathogens and subsequent biofilm maturation [1,2]. Within the protected biofilm matrix, embedded bacteria exhibit altered metabolic phenotypes, enhanced immune evasion, and a phenotypic recalcitrance to conventional antibiotics that can exceed that of their planktonic counterparts by up to a thousand-fold [3,4]. Current clinical management strategies remain largely inadequate; the dense, negatively charged extracellular polymeric substance (EPS) barrier restricts antibiotic diffusion, while sub-inhibitory localized drug concentrations risk selecting for multidrug-resistant (MDR) strains or superbugs [5,6]. Eradicating these persistent microbial communities on topologically complex or irregular implant surfaces, without disrupting host tissue integration, remains a formidable hurdle in contemporary clinical infection control [2,7].

To circumvent the limitations of systemic or localized antibiotic delivery, surface engineering has shifted toward non-antibiotic, multi-functional antimicrobial platforms [3,4]. Traditional approaches typically rely on passive anti-adhesive topographies or active reservoirs that leach heavy metal ions such as Ag^+^, Cu^2+^, Zn^2+^ [2,8,9]. More broadly, a wide variety of functional nanostructured coatings has been engineered using physical/chemical vapor deposition, plasma spraying, sol–gel processing, electrodeposition, hydrothermal synthesis, and layer-by-layer assembly. These fabrication strategies enable precise tailoring of surface roughness, micro-porosity, crystal phase composition, surface energy, and mechanical integrity, directly modulating bacterial docking, biofilm maturation, corrosion resistance, and host cell interactions. In particular, laser-assisted methods—including laser ablation, direct surface texturing, and laser cladding—offer mask-free, highly precise, and spatially controllable avenues to construct hierarchical micro/nanostructures directly on complex implant geometries [10]. By engineering multi-scale physical topographies and surface energy profiles without requiring chemical additives or antibiotic release, laser ablation provides robust passive anti-fouling capabilities while enhancing host tissue integration. Although effective in the acute post-operative phase, these leaching-dependent systems suffer from inherent bottlenecks, including premature payload depletion, localized cytotoxicity to surrounding host cells, and subsequent impairment of tissue repair or osseointegration [11,12]. Furthermore, the inevitable decay of interfacial agent concentrations into sub-lethal windows can inadvertently accelerate resistance selection [13]. Achieving long-term, stable, and on-demand infection control thus demands smart interfaces capable of dynamically disrupting bacterial survival without relying on exhaustible chemical payloads [2].

Piezoelectric nanocoatings offer a compelling paradigm by exploiting force-to-electricity coupling to generate localized, stimulus-responsive therapeutic effects [14,15]. Unlike photothermal or photocatalytic platforms—which are fundamentally constrained by the severe attenuation of light through deep anatomical tissues and the concomitant risk of localized thermal damage—piezoelectric systems leverage ubiquitous, non-invasive mechanical energy [3,4,16]. Ubiquitous physiological forces, such as blood flow shear, joint friction, respiratory vibrations, and mastication, as well as externally applied low-intensity pulsed ultrasound, can serve as the energetic trigger [4,14]. Upon mechanical deformation, these coatings induce transient charge separation and generate surface-bound electric fields at the nano-interface [15]. By coupling inorganic ceramics for high electromechanical response with organic polymers for structural flexibility, hybrid nanocomposites can be tailored to achieve optimal mechanical compatibility and biosafety [4,17,18]. Importantly, these mechanical-to-electrical transduction interfaces exert a multifaceted assault on pathogenic microenvironments. Beyond direct electro-mechanical disruption of bacterial cell membranes, the generated interfacial potential drives localized reactive oxygen species (ROS) production, disrupts bacterial metabolic pathways, and interferes with microbial density-dependent quorum sensing systems. Concurrently, these electrical cues can favorably modulate the peri-implant immune microenvironment, promoting anti-inflammatory macrophage polarization and host tissue integration.

Several recent reviews have summarized broad categories of antibacterial implant coatings, piezoelectric biomaterials, or stimulus-responsive antimicrobial strategies [2], most existing studies either broadly survey piezoelectric materials within tissue regeneration and cancer nanomedicine or focus on static antimicrobial surfaces without systematically addressing piezoelectric nanocomposite coatings as dynamic, anti-infective bio-interfaces for implants [19,20]. In particular, the mechanistic continuum connecting coating micro-architecture, mechanical-to-electrical energy transduction, physical/chemical biofilm matrix disruption, and localized peri-implant immunomodulation remains insufficiently integrated [7]. Furthermore, the distinct application-specific requirements of orthopedic prostheses, dental implants, and skin/wound biomaterials are rarely evaluated within a unified comparative framework [21]. Therefore, a dedicated review is needed to clarify how dynamic piezoelectric nanocoatings transcend conventional passive anti-adhesive, ion-releasing, photothermal, or photocatalytic coatings, while establishing a holistic roadmap detailing their fundamental mechanisms, niche-specific clinical advantages, and translational bottlenecks in durable implant-associated infection control.

**Figure 1:**
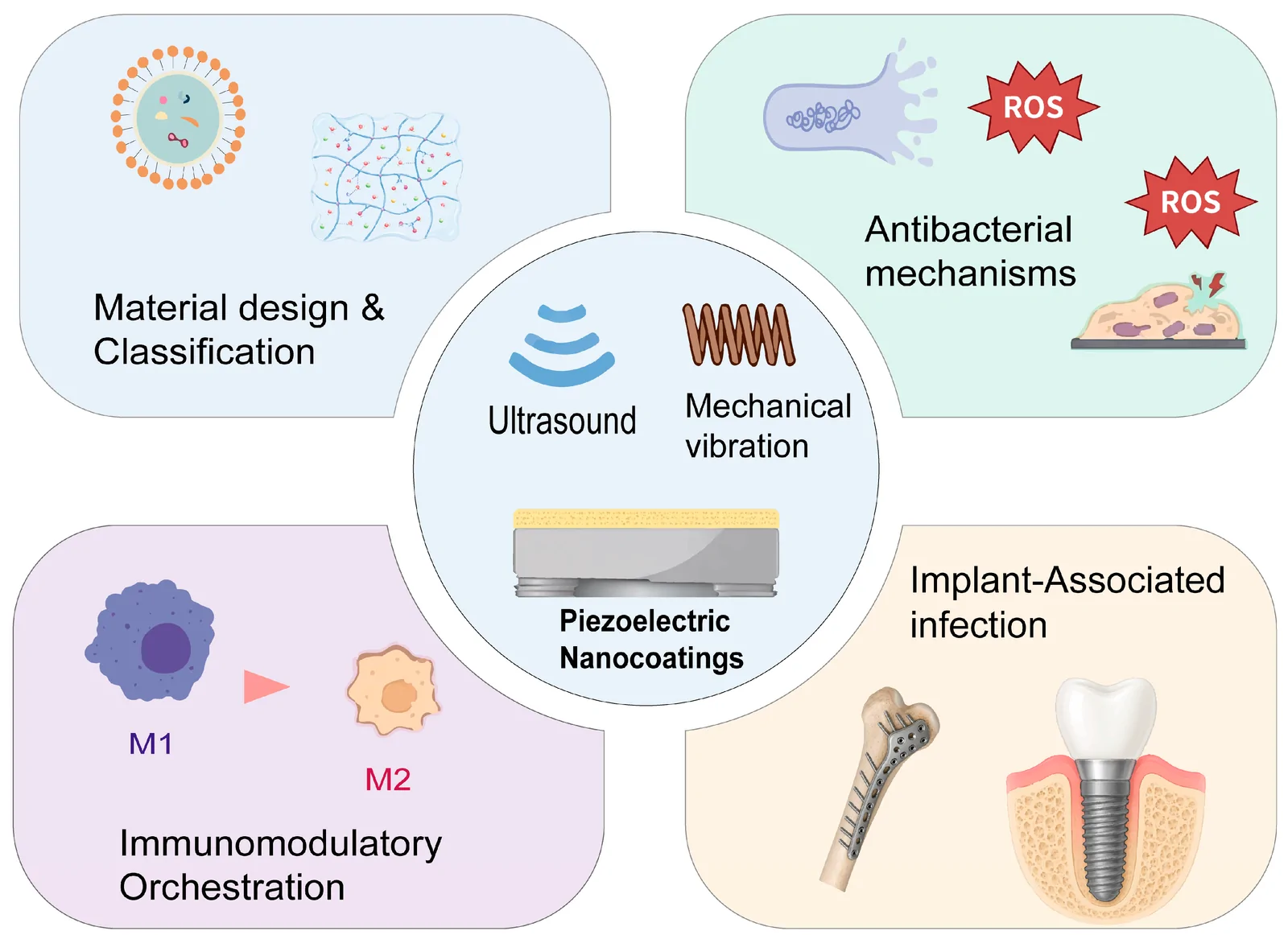
Material design, antibacterial mechanisms, and clinical applications of piezoelectric nanocomposite coatings for the prevention and treatment of implant-associated infections. *Some biological graphic elements were obtained from BioGDP* [22].

This review provides a critical appraisal of piezoelectric nanocomposite coatings as a next-generation frontier in implant surface engineering. We systematically evaluate material design strategies, classifying inorganic, organic, and hybrid architectures based on their electromechanical efficiency and interfacial stability. Crucially, we dissect the underlying biophysical and biochemical mechanisms governing electro-stimulated bacterial death, biofilm eradication, and host-immune modulation. Finally, we contextualize their therapeutic efficacy across prominent clinical scenarios—specifically orthopedic implants, dental devices, and advanced wound management—while critically analyzing the technical bottlenecks and translational trajectories required for clinical adoption (Figure 1).

A structured, transparent literature search was conducted to identify relevant studies on piezoelectric nanocomposite coatings for implant-associated infection control. Primary electronic databases searched included Web of Science, PubMed, Scopus, and Google Scholar. The search string utilized Boolean operators combining thematic keyword groups: “piezoelectric coating”, “piezoelectric nano-composite”, “nanostructured coating”, “implant-associated infection”, “antibacterial coating”, “biofilm”, “reactive oxygen species”, “quorum sensing”, “immune modulation”, “orthopedic implant”, “dental implant”, and “wound dressing”. To ensure exhaustive coverage of inorganic, organic, and hybrid material configurations, specific material descriptors—including “BaTiO_3_”, “ZnO”, “PVDF”, “PVDF-TrFE”, “BiFeO_3_”, and “piezoelectric polymer”—were systematically incorporated. The selection focused primarily on peer-reviewed, English-language research articles and authoritative reviews published over the past decade, while earlier landmark studies were retained to establish foundational principles of piezoelectricity, biofilm architecture, and bio-interface mechanics.

The literature was systematically evaluated according to pre-defined criteria. Studies were included if they reported piezoelectric materials or coatings demonstrating documented antibacterial, antibiofilm, or immunomodulatory activity; investigated nanostructured or nanocomposite surface coatings applicable to biomedical implants or devices; provided clear mechanistic insights into mechanically or acoustically triggered charge generation, ROS production, membrane depolarization, quorum sensing interference, or host immune cell regulation; or targeted clinically relevant applications such as orthopedic hardware, dental implants, vascular/urinary catheters, or wound-contacting biomaterials. Studies were excluded if they focused exclusively on bulk piezoceramics or energy-harvesting devices without relevance to surface coatings or biomedical interfaces; lacked adequate material characterization or quantitative biological assays; were unrelated to infection management or tissue integration; or existed only as unreviewed preprints, non-peer-reviewed conference abstracts, or non-English publications. The final literature portfolio was screened based on thematic alignment, methodological rigor, mechanistic depth, and direct relevance to evaluating the translational viability of smart piezoelectric nanocoatings.

## 2. Material Design and Classification of Piezoelectric Nanocomposite Coatings

The piezoelectric effect originates from the non-centrosymmetric crystalline lattices of specific functional materials, enabling a direct, reversible transduction between mechanical and electrical energy [23]. Under external mechanical stressors—such as compression, tension, or shear deformation—the relative displacement between positive and negative charge centers within the crystal unit cell induces spontaneous dipole polarization. This structural distortion manifests as a macroscopic accumulation of surface charges and the subsequent establishment of a transient, localized piezoelectric potential without requiring external power supplies or hardwired connections [24]. Beyond its widespread adoption in miniaturized electromechanical sensors, energy harvesters, and catalysis, this force-to-electricity coupling holds profound significance at the bio-interface [25]. Endogenous bioelectricity is a fundamental orchestrator within living systems [26]. Specialized mammalian tissues, organs, and extracellular matrix structures exhibit intrinsic piezoelectricity that actively modulates vital physiological pathways, including electrotaxis during cell migration, embryonic morphogenesis, and force-driven bone remodeling [17,27,28].

At the implant–host interface, piezoelectric nanocoatings exploit this bioelectric biomimicry by converting ubiquitous physiological kinetic energy or non-invasive external stimuli like low-intensity pulsed ultrasound into on-demand, localized electrical signals [17,29]. Compared to conventional drug-eluting matrices or metal leaching coatings that inherently suffer from payload exhaustion, systemic cytotoxicity, and the risk of resistance selection, piezoelectric interfaces operate through a responsive, non-consumable physical modality. The dynamic interfacial potential and surface charge density generated at these interfaces directly perturb bacterial transmembrane potentials, disrupt early colonization kinetics, and drive the catalytic generation of ROS to systematically destabilize mature biofilm architectures [3,9]. Crucially, when calibrated within physiological windows, these surface-bound electrical cues concurrently act as biophysical cues that promote host cell adherence, proliferation, and lineage-specific osteogenic differentiation, thereby reconciling pathogen eradication with accelerated host tissue integration [30].

To balance electromechanical conversion efficiency, mechanical compliance, and cytocompatibility at the tissue interface, piezoelectric coatings are strategically categorized into three primary architectural classes: inorganic nanocoatings, organic polymeric coatings, and organic–inorganic hybrid nanocomposites [3,29,31]. Each material system presents distinct characteristics regarding piezoelectric coefficient, elastic modulus matching, interfacial adhesion strength, and long-term chemical stability under complex physiological environments. Dissecting the design and structural classification of these distinct configurations, together with a critical comparison of their antibacterial efficacy, ROS-generation efficiency, biocompatibility, long-term polarization stability, durability, manufacturing scalability, and translational readiness, is essential for understanding their targeted antibacterial mechanisms and guiding the development of clinically viable, infection-resistant, bio-integrative smart implant interfaces.

### 2.1. Inorganic Piezoelectric Nanocoatings

Inorganic piezoelectric nanocoatings primarily comprise ceramic or crystalline frameworks, including lead zirconate titanate (PZT), barium titanate (BaTiO_3_), zinc oxide (ZnO), potassium–sodium niobate, and polarized hydroxyapatite variants [21,27]. Although PZT possesses superior electromechanical coupling, its intrinsic lead toxicity restricts clinical translation due to severe cytocompatibility and environmental hazards [32]. Non-toxic inorganic alternatives exhibit high electromechanical conversion efficiency and robust chemical stability, generating localized surface charge density or inducing sharp potential barriers upon mechanical deformation to arrest microbial colonization and biofilm propagation. In anti-infective applications, these inorganic interfaces exert their antibacterial efficacy through the localized generation of electric fields, oxidative stress, and direct physical membrane damage.

#### 2.1.1. Titanate-Based Piezoelectric Coatings

Titanate ceramics represent the cornerstone of inorganic bio-piezoelectrics [27]. Tetragonal barium titanate (BaTiO_3_, BTO) is widely used in implant engineering due to its exceptional piezoelectric coefficient and good biocompatibility [33]. Under ultrasonic excitation, BTO establishes an internal electric field that drives sonocatalytic water splitting and ROS generation [34,35].This sonomechanical-to-chemical transduction has been engineered for antibacterial applications, where gold-nanoparticle-loaded BTO nanocubes exert powerful sonodynamic bactericidal effects and accelerate tissue granulation under mechanical stimulation [36]. Mechanical deformation of BTO surfaces generates sharp localized potential barriers that depolarize bacterial cell membranes, disrupt metabolic electron transport, and impede biofilm nucleation [37]. Multi-element co-doping strategies further optimize these crystalline structures to enhance output [27].

Integrating BTO with polymeric matrices, hydroxyapatite, or metallic implant surfaces balances electromechanical transduction with interface stability. Wu et al. [38] designed a silver-coated barium strontium titanate (BST-Ag) nanorod array. This topology pairs mechanical puncturing with silver ion release and piezoelectric polarization to achieve rapid, multi-modal bactericidal action while simultaneously providing electro-biophysical cues to accelerate osteoblast proliferation. Similarly, substituting titanium with copper (Cu^2+^) within the BTO lattice enhances the piezoelectric output while exploiting copper’s intrinsic angiogenic and osteogenic pathways to accelerate bone defect healing [39]. To address highly resilient pathogens, Sun et al. [40] utilized an oxygen-deficient BaTiO_3_ nanorod array functionalized with L-arginine (LA). This ultrasonically responsive hybrid coating leverages oxygen vacancies to maximize piezoelectric-catalytic efficiency, eradicating methicillin-resistant *Staphylococcus aureus* (MRSA) via synergistic sonocatalytic oxidative stress and nitric oxide-mediated immunotherapy. Meanwhile, sulfur-doped barium titanate (SDBTO) sonocatalysts incorporate tailor-made oxygen vacancies to enhance charge-carrier separation under ultrasound, successfully clearing *Staphylococcus aureus* (*S. aureus*) biofilms and promoting the osteogenic differentiation of human bone marrow mesenchymal stem cells (hBMSCs) within infectious bone defects [41]. Despite these advancements, the brittleness of monolithic titanate ceramics and their poor interfacial adhesion to metal substrates remain engineering bottlenecks, accelerating the shift toward lead-free, flexible hybrid coatings.

#### 2.1.2. Zinc Oxide-Based Piezoelectric Coatings

ZnO is a non-centrosymmetric wurtzite semiconductor that uniquely fuses intrinsic antimicrobial toxicity with piezoelectric and semiconducting properties [42,43]. This trifecta enables ZnO-based coatings to achieve highly efficient contact-killing alongside stimulus-responsive eradication [17,43]. Tailoring ZnO into nanoarrays or nanowire topologies on titanium, magnesium alloys, or stainless steel significantly amplifies both the localized piezoelectric polarization and mechanical membrane disruption [3,44]. For example, incorporating potassium sodium niobate within a Poly(lactic-co-glycolic acid) (PLGA) framework yields a composite scaffold that synergizes piezoelectrical stimulation with zinc ion (Zn^2+^) release under ultrasound, driving targeted immunomodulation and pathogen clearance in osteomyelitis models [45]. Similarly, Marco et al. functionalized surface-nanostructured ZnO platforms with graphene oxide (GO) or Poly(2-hydroxyethyl methacrylate) (pHEMA), optimizing ultrasound-triggered ROS generation and cytocompatibility for multi-modal tissue engineering and antibacterial applications [46]. In vivo, smart ZnO-Poly(vinylidene fluoride-co-hexafluoropropylene) (ZnO-PVDF-HFP) piezoelectric-coated catheters activated their antibiofilm properties on demand under ultrasonic stimulation, successfully combatting catheter-associated urinary tract infections without localized tissue toxicity [47]. Beyond therapeutics, flexible ZnO nanoarrays utilize strain-induced piezoelectric polarization to modulate interfacial band structures, dramatically elevating the sensitivity of electrocatalytic sensors for real-time hydrogen peroxide (H_2_O_2_) biochemical detection [48]. However, the translation of pure ZnO is limited by its rapid, unregulated dissolution in physiological fluids, where excessive Zn^2+^ accumulation induces cytotoxicity, necessitating precise structural encapsulation to control ion-release kinetics [49].

#### 2.1.3. Niobate-Based Lead-Free Piezoelectric Coatings

Alkaline niobate perovskites represent the premier lead-free alternative for high-output bio-piezoelectrics, including potassium–sodium niobate, potassium niobate (K_0.5_Na_0.5_NbO_3_, KNN), sodium niobate (NaNbO_3_), and lithium niobate (LiNbO_3_) [50]. Under mechanical strain, niobate interfaces project robust surface polarization charges and local electric fields that alter the interfacial electrochemical microenvironment, rendering the surface hostile to initial bacterial docking and biofilm aggregation [51,52]. Specifically, positively polarized KNN surfaces actively reduce *S. aureus* viability while providing an electro-inductive microenvironment that accelerates the adhesion, migration, and proliferation of bone marrow mesenchymal stem cells (BMSCs) [53]. Additionally, niobate coatings possess exceptional chemical inertia and corrosion resistance; for instance, nanoporous NaNbO_3_ coatings deposited on exhibit 316L stainless steel drastically reduce surface roughness and maximize hydrophilicity, shielding the substrate from acid-induced corrosion while promoting host cell migration [54].

Advanced engineering of heterojunctions has expanded their therapeutic field. Incorporating a bismuth-doped sodium niobate/cerium oxide piezo-heterojunction with oxygen vacancies into polyetheretherketone (PEEK) enables ultra-efficient ultrasound-driven ROS generation, resulting in rapid biofilm debridement, anti-inflammatory macrophage polarization, and synchronized osteogenic, vascular, and neural regeneration in infected bone defects [52,55]. Furthermore, integrating KNN into Poly(L-lactic acid) (PLLA) nanofibers significantly upgrades the composite’s electromechanical output, promoting osteogenesis and angiogenesis under low-intensity pulsed ultrasound (LIPUS) [56]. Despite these performance benchmarks, niobate implementation in implant infection control is constrained by high sintering temperatures, poor interfacial bonding to curved metallic implants, and a critical need for rigorous long-term in vivo biophysical stability and degradation assessments [50].

#### 2.1.4. Natural and Biomimetic Piezoelectric Nanocoatings

Emulating the intrinsic bioelectricity of native bone matrix has driven the development of biomimetic and mineral-derived piezoelectric coatings [57]. Hydroxyapatite (HA), the predominant inorganic constituent of bone, exhibits weak endogenous piezoelectricity that can be augmented via crystal orientation tailoring or ion doping [58,59]. For example, prolonging low-temperature hydrothermal synthesis induces a phase transition in HA nanofibers from the centrosymmetric hexagonal phase to the non-centrosymmetric monoclinic phase, substantially enhancing its piezoelectric coefficient while preserving its exceptional osteoconductivity [60]. These biomimetic electrical cues simulate the endogenous streaming potentials of living bone to restrict initial bacterial docking and orchestrate osseointegration. Because monolithic HA possesses limited direct bactericidal efficacy, it is frequently hybridized with ZnO or BTO to achieve bifunctional interfaces [61,62]. Chen et al. [62] engineered a 3D-printed piezoelectric ceramic scaffold containing ZnO-modified BTO with HA, demonstrating broad-spectrum antibacterial performance alongside enhanced osteogenesis and angiogenesis under LIPUS stimulation. Similarly, incorporating HA nanoparticles into Poly(vinylidene fluoride-trifluoroethylene) (PVDF-TrFE) matrices structurally mimics the nanofibrous morphology of collagen–hydroxyapatite networks, accelerating mesenchymal stem cell differentiation and inhibiting microbial accumulation [63]. To optimize this stress-transfer mechanism, Li et al. [64] deployed HA as heterogeneous nucleation sites within PLLA fibers, utilizing precise interfacial topological design to maximize stress concentration and elevate the overall piezoelectric response of the biomimetic matrix.

### 2.2. Organic Piezoelectric Polymer Coatings

Organic matrices capable of electromechanical transduction represent a highly adaptable class of biomaterials, encompassing highly ordered natural biopolymers (e.g., collagen, chitosan, keratin, and cellulose) and synthetic semi-crystalline systems such as Poly(vinylidene fluoride) (PVDF), Poly(L-lactic acid) (PLLA), and polyhydroxybutyrate (PHB) [3,4]. These networks derive their piezoelectric activity from the spatial reorientation of molecular dipoles when subjected to mechanical deformation. PVDF and its copolymer, Poly(vinylidene fluoride-trifluoroethylene) (PVDF-TrFE), stand out as prominent candidates for soft-tissue interfaces and flexible biomedical electronics. Compared to rigid, brittle inorganic ceramics, organic piezoelectric polymers afford structural compliance, reduced mass, excellent processability, and a mechanical impedance that closely mirrors native soft tissues and flexible device form factors [65,66]. The macroscopic electromechanical output of these fluoropolymers is intrinsically tied to their polymorphism, being determined by chain conformation, overall crystallinity, and the relative content of the electroactive, all-trans β phase [67,68].

However, pristine polymer coatings frequently encounter performance bottlenecks, including low native piezoelectric coefficients, unstable long-term bactericidal persistence, and structural degradation when exposed to complex, aggressive physiological fluids [69]. Overcoming these shortfalls necessitates advanced macromolecular engineering and composition refinement to maximize β-phase nucleation [3]. While the incorporation of trifluoroethylene (TrFE) units into PVDF backbone inherently stabilizes the electroactive β-conformation [70], dynamic processing strategies—including electrospinning, spin coating, phase separation, controlled mechanical drawing, and high-voltage corona poling—are widely applied to align molecular dipoles [71]. Hu et al. [72] reported that subjecting electrospun or spin-coated PVDF-TrFE films to an ultra-short, 60 s thermal annealing protocol above their Curie point significantly drives β-phase crystalline enrichment and upgrades the piezoelectric coefficient’s output, establishing an efficient route for high-performance polymer film fabrication. To induce cooperative polarization via structural templating, nanoscale fillers can be integrated into the polymer matrix. For instance, uniformly doping the piezoelectric molecular crystal trimethylchloromethylammonium-cadmium bromochloride (TMCM-CdBrCl_2_) into PVDF-TrFE successfully catalyzes β-phase crystallization and elevates the electromechanical profile [73]. Similarly, Liao et al. [74] synthesized a robust Cu_3_B_2_O_6_/PVDF (CBO/PVDF) hybrid film that incorporates a piezocatalyst into the fluoropolymer matrix, yielding exceptional interfacial stability and enhanced charge-generation kinetics.

When applied as functional bio-interfaces, organic piezoelectric polymers generate localized electrical potentials in response to anatomical movements or external ultrasound, effectively disrupting early microbial docking and blocking biofilm maturation [75]. This mechanical flexibility expands their utility into highly dynamic environments, such as advanced wound care systems, indwelling catheters, wearable physiological monitors, and tissue engineering scaffolds [3]. Regarding electrospun PVDF and elastomeric PVDF, thermoplastic polyurethane (PVDF:TPU) nanofiber meshes, for instance, exert potent bactericidal pressure under cyclic mechanical stretching. The resulting transient surface voltages and local electric fields actively suppress microbial replication rates, with the highly deformable PVDF:TPU blend demonstrating superior antibiofilm efficiency over pristine PVDF [76]. Multifunctional integration can introduce complementary therapeutic modalities into these responsive polymer architectures. Hybrid PVDF/MXene nanofiber membranes combine self-powered, high-sensitivity kinetic sensing with on-demand near-infrared (NIR) photothermal antibacterial properties, serving as an integrated platform for real-time motion tracking and contactless, thermal pathogen eradication [77]. Harnessing ultrasound activation to drive interfacial bioelectricity, Zheng et al. [78] decorated vascular stents with anisotropic piezoelectric nanofibers to boost endothelial cell adhesion, migration, and proliferation. Concurrently, the ultrasound input drives the precise, on-demand elution of encapsulated rivaroxaban, which suppresses the coagulation cascade to minimize thrombotic risks. For advanced wound management, electrospun composite meshes combining PVDF, ZnO nanoparticles (ZnO-NPs), and chitosan (CS) display enhanced electromechanical output under LIPUS. This interactive system coordinates membrane-disrupting antimicrobial toxicity with accelerated host cell migration and microvascular angiogenesis, driving rapid dermal repair with minimal healthy tissue impairment [79,80].

Parallel to synthetic fluoropolymers, PLLA has emerged as a premier biodegradable and cytocompatible piezoelectric substrate for transient or resorbable implants. Electrospun PLLA nanofiber membranes can be engineered with highly tunable piezoelectric coefficient values to align with the specific biomechanical demands of different tissue beds; during in vivo regeneration of critical-sized mandibular defects, these electro-inductive scaffolds stimulated exceptional cellular proliferation rates and bone healing [81]. Utilizing a combined physical–electrical mechanism, self-powered biodegradable Poly(L-lactic acid) (PLLA) membranes unify mechanical pore sieving with continuous electrostatic adsorption to immobilize both airborne particulate matter (PM) and migrating bacterial pathogens, leveraging stable surface potentials to maximize capture efficiency [82]. For structural implant modifications, PLLA matrices can be augmented using zolaphosphonic acid (ZA) as a heterogeneous nucleating agent to boost the localized piezoelectric response. This design establishes a sustained, porous electro-inductive microenvironment that autonomously guides osteogenic lineage differentiation while concurrently suppressing osteoclastic bone resorption via the controlled eluting of ZA, ensuring long-term interfacial stability [83]. Finally, hybrid nanofibrous PLLA scaffolds modified with nano-hydroxyapatite (nHA) further capitalize on this biomimetic synergy to accelerate osteoblast proliferation and surface biomineralization, presenting a viable non-antibiotic horizon for bone infection management [84].

### 2.3. Organic–Inorganic Nanocomposite Strategies

Monolithic inorganic piezoelectric coatings yield superior piezoelectric coefficients and broad operational bandwidths, enabling strong electro-inductive signals. However, their intrinsic brittleness, elevated elastic modulus mismatch, and sub-optimal long-term histocompatibility restrict their integration into compliant implants [85]. Conversely, pristine organic piezoelectric polymers exhibit exceptional mechanical compliance and benign cytocompatibility, yet their suppressed electromechanical transduction coefficients fail to meet the thresholds required for high-efficiency electrical stimulation or targeted antimicrobial therapies. To circumvent these mutual limitations, hybridizing inorganic and organic constituents into integrated organic–inorganic nanocomposites has emerged as a premier engineering strategy. This paradigm successfully bridges the high polarization output of inorganic ceramic/semiconductor fillers with the flexibility, structural resilience, and biocompatibility of the host polymer matrix, achieving a synergistic optimization of interfacial mechanics, biosafety, and localized electrical output [21,86].

A diverse library of hybrid organic–inorganic architectures has been deployed to coordinate concurrent pathogen clearance and tissue regeneration. For example, hydroxyapatite–potassium sodium niobate–chitosan (HA-KNN-CSL) composites have been processed into hydrogels and superporous scaffolds. Within this architecture, lead-free KNN serves as piezoelectric component, while the chitosan and HA components bolster interfacial cytocompatibility, yielding excellent fluid retention and broad-spectrum antimicrobial efficacy against *S. aureus* and *Candida albicans* [86]. Blending PVDF with BaTiO_3_ nanoparticles or coupling PLLA with ZnO to form composite nanofibrous matrices dramatically elevates the macroscopic piezoelectric profile beyond that of pure organic frameworks [87,88], largely because the inclusion of high-permittivity BaTiO_3_ nanostructures modulates the dielectric constant of the surrounding PVDF matrix [89]. Leveraging multi-modal stimuli, Dai et al. [90] developed a flexible piezoelectric–optoelectronic composite film based on PVDF-TrFE/ZnO/TPP, which synchronizes photodynamic action with piezocatalysis to accelerate wound closure. Under simultaneous mechanical deformation and light irradiation, the platform generates a localized electric field that cascades to mutually amplify the therapeutic effects.

For advanced biochemical remediation, exfoliated molybdenum disulfide/Poly(vinylidene fluoride)(E-MoS_2_/PVDF) microcapsules utilize bubble-driven piezoelectrocatalysis to elevate local dissolved oxygen levels and drive the accelerated degradation of residual antibiotic payloads [91]. To accommodate anatomically complex interfaces, a Poly(vinyl alcohol)/Poly(vinylidene fluoride) (PVA/PVDF) composite hydrogel engineered with an osteochondral bilayer structure highlights the structural adaptability of hybrid systems [92]. In this configuration, Ag nanowires are embedded within the mimetic cartilage layer to template the electroactive β-PVDF phase—amplifying polarization output and providing contact-killing antimicrobial protection—while nano-hydroxyapatite (nano-HA) is segregated to the mimetic bone layer to elevate the compressive modulus and stimulate osteogenic differentiation.

Translating these hybrid configurations into clinical practice requires overcoming distinct engineering hurdles, notably nanoparticle agglomeration within the polymer host, weak interfacial covalent bonding, compromised long-term mechanical reliability under fatigue, and challenges in scaling up reproducible manufacturing. Future material design must prioritize the optimization of interfacial stress transfer via surface functionalization, secure chemical and electrical stability in vivo, and balance dynamic piezocatalytic output with long-term cytocompatibility. Resolving these bottlenecks positions the organic–inorganic nanocomposite strategy as a premier trajectory for fabricating next-generation, responsive, and infection-resistant biomaterial interfaces.

Representative piezoelectric antibacterial materials are systematically summarized in Table 1.

Although diverse piezoelectric nanocoatings exhibit clear antibacterial efficacy, their clinical potential is fundamentally governed by distinct material-dependent trade-offs among energy conversion, mechanical compatibility, biological safety, and processability [3].

Inorganic piezoceramics (BaTiO_3_, ZnO, KNN) deliver the highest piezoelectric output, leading to rapid interfacial charge separation and robust ROS generation under acoustic or mechanical excitation [35,38]. This yields rapid bacterial membrane disruption and high short-term antibiofilm efficiency. However, their translational momentum is hindered by intrinsic mechanical brittleness, elastic modulus mismatch with bone/soft tissues, risk of coating delamination, and potential ion-mediated cytotoxicity, such as Zn^2+^ leaching or non-degradable ceramic debris accumulation [2,42,48,51].

Conversely, organic piezopolymers (PVDF, PVDF-TrFE, PLLA) provide excellent mechanical compliance, bio-inertness, coating conformity, and scalable processability (electrospinning, 3D printing), making them ideal candidates for flexible catheters and dynamic tissue interfaces [72,81,97]. Nevertheless, their inherently lower piezoelectric coefficients limit standalone ROS generation under physiological stress, often requiring surface nanostructuring or catalytic filler integration to achieve clinically relevant antibacterial performance. Furthermore, long-term exposure to physiological moisture can trigger dipole relaxation and phase instability, gradually dampening polarization output [71,72,80].

Organic–inorganic hybrid nanocomposites (BaTiO_3_/PVDF, ZnO/PLLA) currently represent the most balanced paradigm. By embedding high-output piezoceramic fillers within flexible polymeric matrices, these platforms simultaneously optimize interfacial charge transport, ROS generation, mechanical flexibility, and cytocompatibility [87,88]. Despite these clear synergistic benefits, hybrid systems face distinct translational hurdles, including nanofiller aggregation, batch-to-batch manufacturing variation, unresolved long-term filler degradation pathways, and stringent regulatory scrutiny governing multifunctional medical devices [2,90,92].

Currently, the field remains predominantly at the proof-of-concept stage, characterized by short-term in vitro assays or small-animal models. Advancing piezoelectric coatings toward clinical reality requires standardized head-to-head evaluations using clinical multidrug-resistant isolates, polymicrobial biofilms, unified acoustic/mechanical excitation parameters, standardized ROS quantification protocols, and rigorous long-term polarization/adhesion testing in large-animal models.

## 3. Antibacterial Mechanism of Piezoelectric Coatings

**Figure 2:**
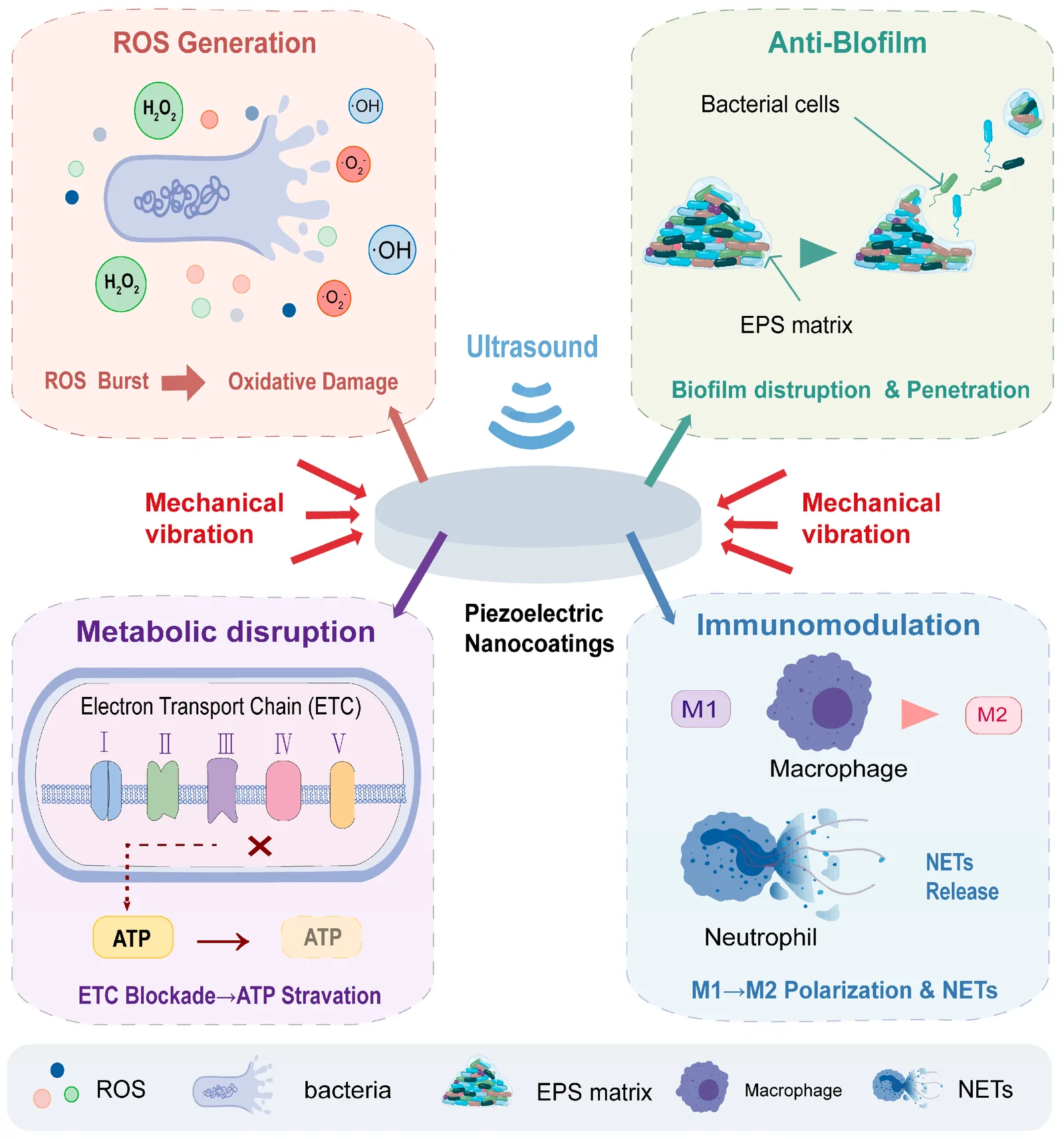
Antibacterial and immunomodulatory mechanisms of piezoelectric nanocoatings against implant-associated infections. The piezoelectric effect induces a burst of reactive oxygen species (ROS), including ·OH, ·O_2_^−^, and H_2_O_2_, which attack bacterial membranes and cause oxidative damage; nanoparticles penetrate the biofilm and disrupt the extracellular polymeric substance (EPS) matrix, promoting biofilm disassembly; piezoelectricity-induced redox perturbation blocks the bacterial electron transport chain (ETC), resulting in impaired ATP production and metabolic energy depletion; and the implant microenvironment regulates macrophage polarization from the M1 to M2 phenotype, accompanied by neutrophil extracellular trap (NET) release, thereby synergistically promoting inflammation resolution and infection control. This figure summarizes how piezoelectric polarization activation enables anti-infective therapy through oxidative stress, biofilm disruption, metabolic inhibition, and immune modulation. *Some biological graphic elements were obtained from BioGDP* [22].

IAIs present a formidable clinical hurdle, primarily initiated by the persistent colonization and proliferation of pathogenic microbes on the surfaces of indwelling biomedical devices. Pathogens introduced during the perioperative window, reinforced by localized inflammatory cascades, compromise the host–tissue interface and render the peri-implant microenvironment highly susceptible to chronic infection, frequently culminating in mechanical failure, aseptic loosening, and severe postoperative complications [1,3,4]. The infectious microenvironment encompasses not only the invading pathogens themselves but also a localized, altered physiological niche characterized by altered pH, hypoxia, and compromised immune surveillance. Piezoelectric nanocoatings actively counter these challenges by concurrently targeting the structural integrity of the pathogens and modifying their immediate survival environment (Figure 2). The intrinsic electromechanical transduction and micro-biocatalytic properties of these polarized coatings drive the continuous generation of reactive species, generating localized potential barriers that destabilize bacterial cell membranes and intercept both intracellular and extracellular electron transfer vectors. Furthermore, these dynamic coatings exert broad-spectrum antimicrobial activity by disrupting the structural matrix of biofilms, altering bacterial metabolic and bio-energetic pathways, and transmuting the host immune microenvironment from a state of infection-induced paralysis to active clearance [3,99].

### 3.1. Generation of Reactive Oxygen Species

The generation of localized oxidative stress stands as a primary bactericidal pathway engineered into piezoelectric biomaterials. ROS comprise a family of highly unstable, partially reduced oxygen-containing derivatives, primarily including hydrogen peroxide (H_2_O_2_), superoxide anion (·O_2_^−^), singlet oxygen (^1^O_2_), and hydroxyl radical (·OH) [3,100]. Under normal physiological conditions, endogenous ROS function as metabolic byproducts of mitochondrial respiration, participating in cell signaling and homeostatic regulation. During pathogen invasion or other stress conditions, the intracellular accumulation of these radicals can overwhelm the bacterial antioxidant defense network, shifting the localized redox equilibrium toward destructive oxidative stress. When ROS concentrations surpass the enzymatic clearance thresholds of the cell, they induce irreversible lipid peroxidation within cell membranes, cause oxidative degradation of functional proteins, and trigger extensive nucleic acid fragmentation, resulting in rapid metabolic collapse and cell death [101].

Within piezoelectric coatings, mechanical stimulation drives the spatial separation of photogenerated or mechanically excited electron–hole pairs across the crystalline lattice, steering these highly reactive charge carriers toward the material–liquid interface to participate in surface redox chemistry. In the presence of ambient water and dissolved oxygen molecules, these separated charges catalyze cascade reactions that yield substantial yields of ·OH, ·O_2_^−^, and H_2_O_2_. This stress-responsive radical generation strategy has been cross-linked with external energy modalities, including photodynamic therapy (PDT), sonodynamic therapy (SDT), enzymodynamic therapy, and chemodynamic therapy (CDT) [102].

For example, TiO_2_/Bi_2_WO_6_ heterojunction arrays engineered as piezoelectric implants respond synergistically to NIR light and endogenous mechanical forces [103]. Under NIR irradiation, the heterojunction amplifies photon capture within the tissue-transparent near-infrared window by introducing targeted oxygen vacancies. Concurrently, it accelerates radical generation via charge carrier separation driven by an intrinsic electric field (IEF), emitting concurrent electrical and chemical signals that guide osteogenic lineage differentiation and implant osseointegration [103].

As a clinical mechanical input, ultrasound (US) waves penetrate deeply into dense tissue structures, triggering localized acoustic vibration and hydrodynamic cavitation effects that amplify mechanical stress at the bacterial interface [104]. Acoustic energy propagates through the peri-implant zone, forcing the embedded microbes to experience severe shear stress and localized radical bursts [105]. This principle is exploited by gold-nanoparticle-loaded barium titanate (Au@BTO) nanocubes. Under acoustic deformation, the BTO core undergoes a lattice shift to generate a robust internal polarization potential [36]. The spatial band bending at the Au-BTO interface establishes a sharp Schottky barrier that traps migrating electrons and systematically suppresses electron–hole recombination, optimizing the lifetime of surface charge carriers to drive the continuous conversion of H_2_O and O_2_ into bactericidal ·OH and ·O_2_^−^ radicals [33].

### 3.2. Inhibition of Bacterial Adhesion and Biofilm Formation

A biofilm represents a highly structured, sessile microbial consortium that develops when planktonic bacteria anchor to an abiotic surface and secrete a protective matrix of EPS [106]. This self-assembled EPS is composed of extracellular polysaccharides, structural proteins, lipids, and extracellular DNA (eDNA), and functions as a dense physical and chemical barrier that deactivates conventional antibiotics via molecular adsorption, steric hindrance, or enzymatic degradation. Furthermore, the restricted diffusion gradients established within mature biofilms generate distinct localized microenvironments characterized by hypoxia, nutrient deprivation, and hyperacidity [3,107,108]. This hostile niche forces the embedded bacteria into a state of metabolic dormancy or down-regulated replication, giving rise to antibiotic-tolerant persister cells and accelerating horizontal gene transfer pathways that amplify drug resistance up to 1000-fold relative to free-living planktonic populations. Once initial microbial attachment transitions into a mature, multi-layered biofilm, the infection becomes highly resilient against both systemic pharmacology and host immune clearance [108,109]. Piezoelectric nanocoatings intercept this pathogenic progression through a multi-stage chronological defense strategy:

1. Interdiction of early adhesion: Transient surface charges generated by continuous piezoelectric polarization modulate the material’s surface energy and alter the initial electrostatic attraction between the substrate and the negative cell walls of migrating bacteria. For example, BTO-containing piezoelectric hydrogels subjected to mechanical deformation generate localized surface charge densities that actively down-regulate the transcription of the bacterial surface polysaccharide biosynthesis gene (*porP*) and the *fimA* gene, which encodes the major structural subunit of bacterial fimbriae, thereby arresting early docking and microcolony nucleation [110].
2. Disruption of proliferation and transmembrane ion balance: During the critical phases of microbial replication and early cluster expansion, the localized micro-electric fields projected by the deformed piezoelectric crystals alter the trans-membrane potential of adjacent bacterial cells. This electrical perturbation disrupts voltage-gated ion channels and collapses essential transmembrane proton gradients, pairing with mechanical shear stress to impede cell division and slow down biofilm propagation.
3. Deep biofilm penetration: To eradicate established biofilms that have already formed an initial structural barrier, augmenting the active penetration capability of the biomaterial is essential. This was addressed by developing a mushroom-shaped Janus nanomotor utilizing a piezoelectric tetragonal barium titanate (T-BTO) core, an asymmetric polydopamine (PDA) coating, and peripheral L-arginine functionalization [92]. When introduced into an infected microenvironment, this nanomotor utilizes endogenous H_2_O_2_ to drive the unilateral catalytic generation of nitric oxide (NO), generating an asymmetric chemical propulsion vector that achieves self-propelled motility and enhanced biofilm penetration. Under concurrent ultrasonic stimulation, the T-BTO core undergoes internal polarization to generate a radical burst via the piezoelectric effect, acting in synergy with the co-delivered NO payload to debride the EPS matrix and eradicate embedded drug-resistant persisters.
4. EPS matrix degradation: During the final stages of biofilm maturity, piezo-catalytically generated ROS trigger powerful oxidative cleavage on the structural macromolecules within the EPS framework. By fragmenting long-chain polysaccharides, denaturing structural proteins, and breaking down eDNA networks, these radicals increase the porous network density of the matrix, effectively dismantling the physical barrier and allowing deep diffusion of complementary antimicrobial factors [107]. Concurrently, the localized oxidative pressure disrupts bacterial quorum sensing (QS) circuits by chemically degrading autoinducer signaling molecules, blocking inter-bacterial communication and preventing collective colonization.

**Figure 3:**
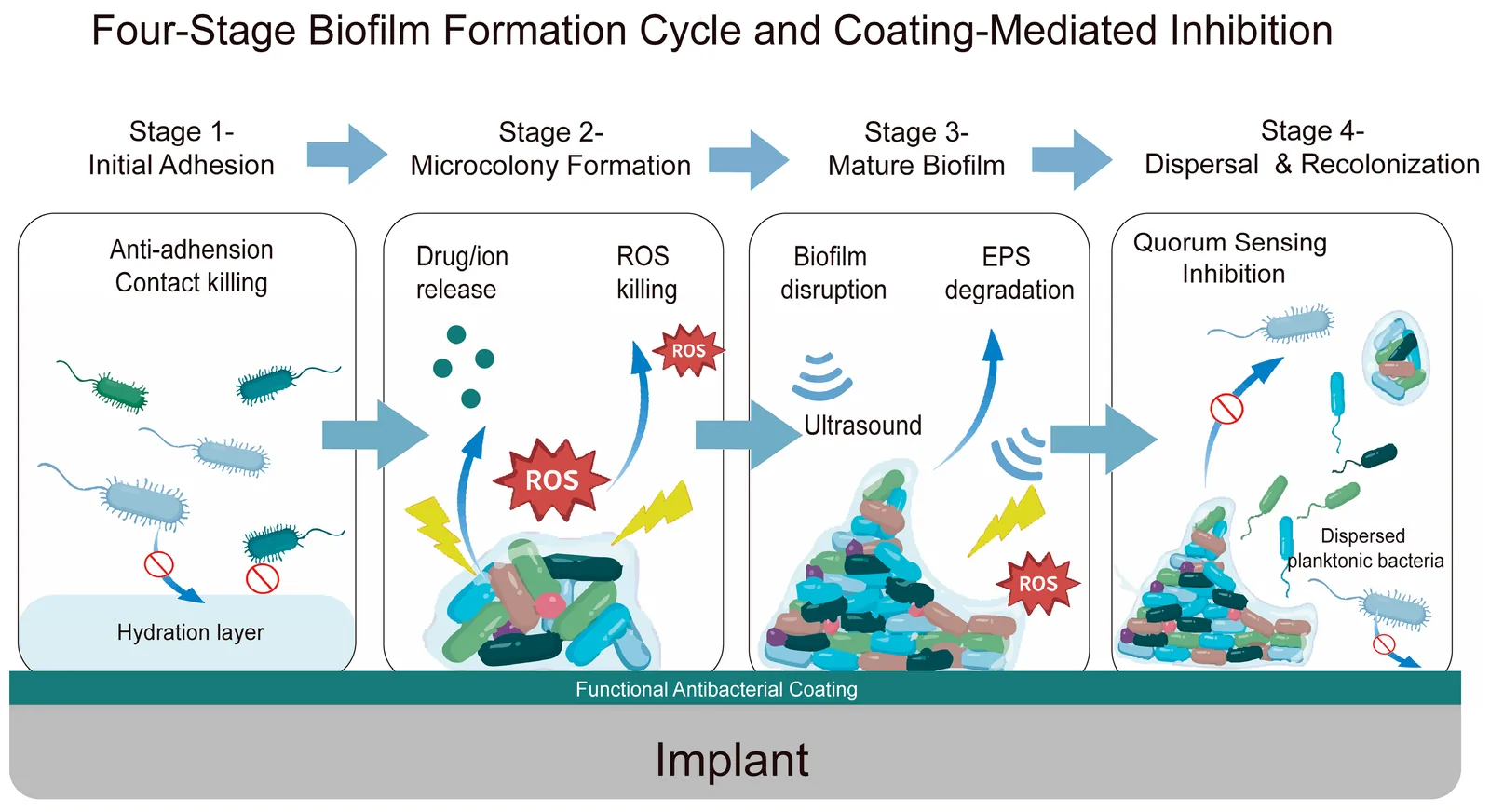
Schematic illustration of the four-stage biofilm formation cycle and coating-mediated inhibition on implant surfaces. During initial adhesion, a hydrated anti-adhesive layer and contact-killing activity suppress bacterial attachment. During microcolony formation, released drugs/ions and ROS inhibit bacterial proliferation. In mature biofilms, ultrasound-assisted biofilm disruption, EPS degradation, and ROS generation promote biofilm eradication. During dispersal and recolonization, the functional antibacterial coating inhibits bacterial regrowth and reduces recolonization. *Some biological graphic elements were obtained from BioGDP* [22].

By simultaneously intervening across the adhesion, proliferation, and collective colonization checkpoints, piezoelectric nanocoatings continuously reduce the risk of mature biofilm development—an attribute required for securing long-term implant integrity (Figure 3).

### 3.3. Disruption of Bacterial Metabolism and Bio-Energetics

Polarized bio-interfaces deploy localized potential barriers and micro-electric fields to induce bio-energetic collapse within adjacent bacteria, primarily by targeting the cell membrane and the integrated electron transport chain (ETC) [3]. Bacterial cell membranes rely on a highly regulated, asymmetric distribution of ions to maintain a stable resting membrane potential, which powers vital substance transport, metabolic homeostasis, and adenosine triphosphate (ATP) synthesis. When an external or strain-induced micro-electric field shifts this electrochemical balance, the structural integrity of the lipid bilayer is compromised, manifesting as irreversible membrane depolarization, unregulated poration, and an increase in outer membrane permeability [107]. This physical breach drives the rapid leakage of vital intracellular components, electrolytes, and metabolic intermediates, while concurrently deactivating membrane-bound metabolic enzymes and halting vital nutrient uptake pathways [111].

This mechanism is particularly relevant in the context of antimicrobial resistance. Conventional antibiotics usually act through specific biochemical targets, such as cell-wall synthesis, protein translation, DNA replication, or folate metabolism. Consequently, bacteria readily acquire resistance through target-site mutations, enzymatic drug degradation, efflux pump overexpression, reduced membrane permeability, and horizontal transfer of resistance genes. In contrast, piezoelectric antibacterial coatings operate primarily via localized physical and physicochemical stresses—including membrane depolarization, interfacial micro-electric fields, ROS generation, and respiratory-chain disruption. Because these contact-localized, multi-target mechanisms do not depend on a single drug target interaction or sustained systemic antibiotic exposure, they significantly reduce the likelihood of selecting for classical genetic antibiotic resistance.

To exploit this metabolic vulnerability, Xu et al. [112] designed metal/piezoelectric nanostructures embedded onto the surface of polymer implants. Upon ultrasonic stimulation, these configurations generate micro-scale electrical discharges at the implant–bacteria interface, inducing severe localized oxidative stress that destroys the cell wall of adherent *S. aureus* while driving rapid glucose metabolism depletion—a strategy validated for clearing deep subcutaneous infections without damaging adjacent host tissue. Similarly, a piezoelectric catalytic bioheterojunction (P-bioHJ) composed of bismuth oxyiodide (BiOI) and few-layer MXene structures couples acoustic excitation with unique electronic boundary conditions [113]. This heterojunction responds to ultrasound by generating localized sonoluminescence while utilizing its polarization-matched interface and induced oxygen vacancies to promote ultrafast charge-carrier separation, thereby triggering an immediate free-radical burst. This heterojunction responds to ultrasound by generating localized sonoluminescence while utilizing its polarization-matched interface and induced oxygen vacancies to promote ultra-fast charge carrier separation, triggering an immediate free-radical burst. Crucial to its performance is the direct disruption of the bacterial electron transport chain, which halts inner-membrane energy synthesis and arrests downstream metabolic processing.

Furthermore, investigations by Geng et al. confirm that engineered piezoelectric heterojunctions target specific respiratory enzymes, significantly suppressing the enzymatic kinetics of bacterial complex IV [114]. By blocking downstream electron flux along the respiratory chain, these platforms halt proton translocation across the inner membrane, collapsing the proton motive force required to power F1F0-ATP synthase, thereby inducing absolute bio-energetic starvation within the pathogen. While this dual mode of electric-field interference and metabolic disruption offers a promising non-antibiotic route to manage multidrug-resistant strains, the resistance-related behaviors of bacteria under piezoelectric stimulation remain insufficiently understood. Important knowledge gaps persist regarding whether repeated sublethal piezoelectric stimulation could select for stress-tolerant phenotypes, enhance oxidative-stress defense systems, alter membrane repair pathways, or trigger persister-cell formation. Future studies should systematically evaluate long-term bacterial adaptation, resistance-gene expression profiles, persister dynamics, and treatment efficacy within clinically relevant polymicrobial biofilms and in vivo infection models.

### 3.4. Immunomodulatory Orchestration of Host Innate Immunity

Beyond executing direct bactericidal action, piezoelectric nanocoatings function as active immunomodulatory platforms that tune host innate immune cells within the peri-implant niche. Among these, macrophages act as essential frontline effectors, displaying extreme functional plasticity by shifting dynamically between the pro-inflammatory M1 phenotype—which drives early pathogen clearance—and the anti-inflammatory, reparative M2 phenotype, which coordinates tissue remodeling and matrix deposition [115]. In a classical infection model, mature bacterial biofilms deploy specific virulence factors to paralyze adjacent immune cells, blinding macrophages and blocking their ability to phagocytose or clear the infection, which locks the peri-implant space into a state of chronic, destructive inflammation [107].

Driven by low-intensity ultrasound or anatomical kinetic deformation, piezoelectric nanocoatings project localized surface charge gradients that function as bioelectrical cues to reshape the immune response [104]. Research by Li et al. [116] demonstrates that titanium implant surfaces modified with piezoelectric nanostructures significantly amplify the internal phagocytic capacity of host macrophages against *S. aureus* when acoustically activated. The core mechanism involves the activation of the phosphatidylinositol 3-kinase–protein kinase B (PI3K-AKT) and mitogen-activated protein kinase (MAPK) intracellular signaling pathways. Similarly, ultrasound-activated BTO nanoparticles utilize controlled micro-scale radical generation to alter macrophage and force cytoskeletal realignment, driving classical polarization toward the active M1 phenotype. This shift triggers the robust up-regulation of primary pro-inflammatory markers and bactericidal cytokines—including tumor necrosis factor-α (TNF-α), interleukin-1 β (IL-1β), interleukin-6 (IL-6), and inducible nitric oxide synthase (iNOS)—via coordinated signaling through the PI3K/Akt, MAPK, and FcγR pathways, effectively restoring the host’s innate capacity to engulf and digest biofilm-shielded pathogens [117].

However, sustaining an unattenuated M1 pro-inflammatory environment can induce severe collateral damage, including the inhibition of microvascular angiogenesis, deceleration of tissue granulation, and the acceleration of peri-implant bone resorption [3]. Therefore, after the infection has been initially controlled, the bio-interface must promote a timely transition from the M1 clearing phase to the M2 tissue repair phase to resolve chronic inflammation and support osteogenesis. Advanced piezoelectric frameworks achieve this phase-dependent transition by upregulating arginase-1 (Arg-1), clusters of differentiation 206 (CD206), and anti-inflammatory signaling molecules such as IL-4 and IL-10 during late post-infectious healing [107,118]. Mechanistically, these sustained, low-level electrical signals induce metabolic reprogramming within the host cells, stimulating heightened fatty acid oxidation, amino acid biosynthesis, and mitochondrial biogenesis to supply the energetic payloads required to sustain the M2 reparative phenotype [116].

Concurrently, neutrophils represent an additional vital cellular target for piezoelectric immunomodulation. Han et al. designed a biomimetic piezoelectric–conductive integrated peri-implant gingiva (PiG) architecture by pairing a highly flexible piezoelectric film and a conductive polymer network. Under ultrasonic drive, this platform delivers precise electrical signals that alter the resting membrane potential of adjacent host neutrophils, opening voltage-gated calcium channels to stimulate a rapid Ca^2+^ influx and an internal ROS burst. This intracellular cascade triggers the formation and release of neutrophil extracellular traps (NETs) [119]. These NETs act as physical mesh structures that capture migrating bacteria, degrade circulating virulence factors via bound proteases, and work in synergy with material-generated surface radicals to clear the infection zone, demonstrating that piezoelectric coatings can activate host innate immunity to drive multi-modal pathogen clearance. While these host–material interactions hold clinical promise, establishing precise control over the signal intensity, operational duration, cellular response thresholds, and exact downstream molecular cascades remains an active focus of bio-interface research [3]. The antibacterial efficacy, stimulation conditions, and immunomodulation of piezoelectric nanocoatings are systematically summarized in Table 2.

## 4. Piezoelectric Coatings in Implant-Associated Infection Control

### 4.1. Oral and Dental Implants

Pathogen-mediated colonization of oral implant fixtures represents a complex clinical challenge in dental surface engineering [112]. Dental implants operate within a hostile environment, experiencing continuous exposure to mixed-species salivary microbiomes, chemical fluctuations from food debris, and shear stress variations. This microenvironment favors early bacterial docking and subsequent maturation into dense biofilms. Left unchecked, this progression triggers peri-implantitis, localized soft-tissue necrosis, and osteoclast-mediated alveolar bone resorption, compromising the mechanical stability and survival of the implant fixture. Consequently, developing non-invasive, highly effective, and antibiotic-independent anti-infection strategies is essential for modern dental implant design [1]. Piezoelectric nanocoatings and piezoelectric composite interfaces offer an autonomous solution by generating localized electric fields and surface charges in response to physiological masticatory forces, ultrasonic waves, or micromechanical vibrations. This electromechanical induction drives the continuous generation of ROS that disrupt bacterial membrane structures and inhibit biofilm maturation, providing a self-responsive strategy for dental implant protection [28,123].

Integrating functional embedding metal and piezoelectric nanostructures into polymer dental implants enables targeted pathogen eradication under acoustic activation. The ultrasonic-driven localized piezoelectric effect induces oxidative stress at the implant–bacteria interface, destroying the cell wall of adherent *S. aureus* while causing rapid glucose metabolism depletion. This strategy has been applied to treat endodontic reinfections. For example, embedding metal/piezoelectric nanostructures into Eucommia gum root canal obturation systems achieved effective clearance of persistent intracanal pathogens [112]. This approach can also be used to engineer the complex trans-mucosal and intraosseous zones of titanium fixtures. Constructing piezoelectric/metal heterostructural coatings combats in situ *S. aureus* biofilms in peri-implantitis models through a dual pathway of piezoelectric-catalyzed radical generation and host innate immune activation, while simultaneously improving osseointegration at the bone–implant interface [115].

In addition to mechanical inputs, daily temperature fluctuations can drive surface charge variations via pyroelectric mechanisms. A pyroelectric functional coating synthesized on Ti implants generates localized bioelectrical stimulation in response to daily intraoral temperature shifts. By pairing cold-induced thermal gradients with the pyroelectric effect, this bio-interface activates transmembrane TRPM8 ion channels to trigger Ca^2+^ influx into host stem cells, enhancing early peri-implant bone remodeling [123]. Naturally occurring masticatory movements also provide a regular source of mechanical stimulation to power these coatings. Shi et al. [121] developed an oral motion-driven smart dental implant abutment (SDIA) utilizing a resin-reinforced, high-toughness piezoelectric BTO ceramic composite. This system converts intermittent occlusal pressure into surface charges, driving ROS-mediated bactericidal activity. Concurrently, the SDIA projects surface electrical cues that regulate the host MAPK and PI3K-Akt pathways to accelerate soft-tissue fibroblastic adhesion, achieving coordinated infection control and soft-tissue sealing. Similarly, autonomous piezoelectric dental implants driven by daily occlusal loads convert mechanical strain into stable bioelectric signals, inducing ROS production, guiding macrophage phenotypic shifts toward anti-inflammatory states, and activating osteogenesis-related genes to enhance bone healing [124]. To counter soft-tissue infections and poor epithelial sealing at the gingival margin, researchers have developed biomimetic conductive interfaces for the PiG region combining flexible piezoelectric films with conductive polymer networks [119]. Under ultrasonic stimulation, this interactive network generates bactericidal effects while promoting host cell adhesion. In rat subcutaneous infection models, ultrasonically irradiated PiG-grafted Ti implants effectively cleared *S. aureus* infections, salvaged contaminated fixtures, and enhanced soft tissue integration [119].

### 4.2. Orthopedic Implants

Infections in orthopedic implants, such as joint prostheses and spinal fixation systems, are driven by the rapid adhesion and biofilm formation of pathogens like *S. aureus* on structural metal surfaces [21,125]. This colonization causes localized osteolysis, chronic osteomyelitis, and aseptic loosening, often necessitating revision surgery. Applying polarized coatings to orthopedic implants helps prevent biofilm formation while actively supporting bone integration.

To achieve this, a Ba/Ti-doped perovskite BiFeO_3_-BaTiO_3_ (BFBT) piezoelectric nanoreactor was integrated with hydroxyapatite onto porous titanium bone scaffolds [93]. Under acoustic drive, the TH-BFBT coating projects an intrinsic electric field that drives the generation of ROS and H_2_O_2_, while accelerating the Fe(III)/Fe(II) valence cycle to deliver a cascading piezoacoustic-chemotherapeutic response that clears persistent intraosseous pathogens. Magnetron sputtering has also been used to grow a ZnO-modified coating (TaNTs@ZnO) on the surface of tantalum nanotubes without altering their porous macro-topography [96]. The piezoelectric ZnO phase provides pH-responsive Zn^2+^ release, achieving more than 99% antibacterial efficiency against *S. aureus* and *Escherichia coli* (*E. coli*) by disrupting bacterial membranes and metabolic pathways [96].

Similarly, a zirconium single-atom-doped SrTiZrO_3_/hydroxyapatite (STZO/Hap) heterojunction coating utilizes interfacial polarization to enhance piezocatalytic and sonodynamic activities under ultrasound [122]. This is paired with the immunotherapeutic activity of released Zr^4+^ ions to achieve effective dual-line clearing of Gram-positive and Gram-negative pathogens. For flexible orthopedic applications, PVDF-TrFE films incorporated with the ionic liquid [Emim][HSO_4_] offer anti-biofilm activity against *S. aureus* and *E. coli*, while supporting the adhesion, proliferation, and osteogenic differentiation of osteoprogenitor cells [98]. By converting physical movement into bioelectric signals that mimic the native piezoelectric microenvironment of healthy bone tissue, this flexible system serves as an interactive osteointegrative interface and tissue-engineering scaffold [98].

Furthermore, the copper-containing modified barium titanate (PH-CpBT) coatings grown on 3D-printed polyetherketoneketone (PEKK) bone scaffolds generate piezoelectric hot carriers under ultrasound to drive the Cu^2+^/Cu^+^ valence cycle [126]. This mechanism combines acoustic energy with chemical therapy to generate ·OH, disrupting bacterial membrane homeostasis and inducing a cuproptosis-like death pathway in *S. aureus*. Concurrently, the coating stimulates osteogenesis through copper-ion-mediated angiogenesis and biomineralization driven by calcium and phosphorus ions released from the hydroxyapatite matrix.

Piezoelectric modifications can also directly improve long-term mechanical anchorage. Magnetron-sputtered BaTiO_3_-SrZrTiO_3_ coatings deposited on zirconium alloy implants significantly enhanced the early adhesion, replication, and alkaline phosphatase differentiation of host osteoblasts [94]. Furthermore, BaTiO_3_ coatings synthesized on porous Ti6Al4V scaffolds promote M2 macrophage polarization, establishing an anti-inflammatory microenvironment that supports immunoregulatory osteogenesis in osteoblasts. Complementing these surface coatings, piezoelectric technology can also assist in active physical debridement. Applying piezoelectric ultrasound to infected orthopedic hardware removed mature bacterial biofilms from various metallic and polymeric implant surfaces with significantly higher efficiency than conventional pulsed irrigation, presenting a viable mechanical method for clinical biofilm management [127].

### 4.3. Other Implantable Devices

Beyond dental and orthopedic hardware, indwelling polymeric devices such as catheters are primary vectors for nosocomial infections. Flexible elastomer lines—including epidural, central venous, and urinary catheters—experience high rates of microbial contamination. Pathogenic attachment and biofilm maturation along these polymer walls shield microbes from systemic therapies, increasing the risk of catheter-associated urinary tract infections (CAUTIs) and bloodstream infections [1]. To address this, a ZnO-PVDF-HFP functional coating containing piezoelectric ZnO nanoparticles was applied to the surface of silicone urinary catheters [47]. When activated by external ultrasound, the coating generates localized surface potentials that inhibit bacterial growth and biofilm formation. This on-demand antimicrobial performance can be adjusted by tuning the external acoustic parameters. In animal models, these piezoelectric-coated catheters reduced the incidence of catheter-associated urinary tract infections without inducing systemic cytotoxicity or mucosal inflammation, demonstrating the viability of responsive coatings for flexible medical lines [47].

Piezoelectric coatings can also be used to treat soft-tissue infections in the gastrointestinal tract. Furthermore, Zhao et al. [97] developed a flexible PVDF-TrFE piezoelectric nanofiber film (PNF) as an anti-infective therapeutic patch designed to modulate the electro-microenvironment of biofilms surrounding gastrointestinal wounds. When coupled with external stimulation, the PNF patch accelerates charge transfer kinetics through the EPS of the biofilm. This localized electrical flux breaks down the molecular bonds of the EPS matrix, increasing its porosity and disrupting its structural integrity, which enhances the penetration and efficacy of co-delivered antimicrobial agents at the wound site.

## 5. Summary and Outlook

### 5.1. Summary of Progress and Current Translational Bottlenecks

Piezoelectric nanocoatings have emerged as a highly promising paradigm in functional biomaterials, executing effective pathogen clearance by transducing ambient mechanical energy—including external clinical ultrasound, native musculoskeletal movement, or the continuous hydrodynamic flow of physiological fluids—into localized electrical signals and a cascade of highly reactive chemical species. This dynamic, stress-responsive mechanism stops early bacterial attachment, restricts colony proliferation, breaks down the protective EPS shield of mature biofilms and modulates host immune responses at the implant interface. Compared to traditional systemic antibiotic therapy and leaching-type antimicrobial surfaces, these smart bio-interfaces offer distinct translational advantages, notably non-antibiotic bactericidal kinetics, precise controllability, reduced risk of inducing genetic drug resistance, and the structural capability to be integrated onto complex implant surfaces or flexible indwelling medical lines. Recent architectural engineering of inorganic semiconductors/ceramics, fluoropolymer matrices, and polarization-matched bioheterojunctions has achieved excellent multi-modal antimicrobial performance while simultaneously driving downstream tissue remodeling, accelerated osteoblast differentiation, and phase-dependent host immunomodulation within specialized biomedical niches.

Despite this validation at the bench scale, translating piezoelectric nanocoatings into clinical microbiological and orthopedic interventions requires overcoming several key engineering bottlenecks and multi-dimensional biological challenges:

1. Piezoelectric coatings face severe chemical and physical degradation when exposed to the highly corrosive, humid, and homeostatically regulated in vivo environment. Prolonged exposure to body fluids and body temperature can trigger dipole relaxation within semi-crystalline organic fluoropolymers, causing a gradual decline in their polarization potential and electromechanical transduction efficiency over time, which subsequently compromises their long-term, on-demand bactericidal performance;
2. Traditional heavy-metal formulations like lead zirconate titanate possess high piezoelectric coefficients, but their intrinsic lead toxicity, absolute brittleness, and poor soft-tissue histocompatibility exclude them from long-term clinical implantation. Conversely, benign organic alternatives like PLLA and PVDF match the compliance of native biological tissues but exhibit low piezoelectric coefficients. This structural limitation frequently fails to meet the polarization thresholds required to drive high-efficiency chemical therapies or rapid bone remodeling under weak physiological stimuli;
3. Lead-free piezoceramics and highly engineered organic–inorganic nanocomposites represent a viable trajectory to balance functional output with material safety. However, the long-term biosafety of these mixed configurations remains to be systematically verified. Concerns persist regarding the potential for nanoparticle leaching from the polymer host, the systemic distribution and clearance pathways of non-degradable fillers, and the localized cytotoxicity of accumulation byproducts;
4. Hard-tissue implants are continuously subjected to complex and cyclic loading. Under these conditions, the mismatch in elastic modulus at the coating–substrate interface can induce mechanical fatigue, micro-cracking, or catastrophic delamination. This structural failure not only causes the complete loss of the active piezoelectric antimicrobial functionality but the shed particulate debris can also trigger aseptic loosening or unwanted immune responses.

### 5.2. Future Research Avenues

To advance piezoelectric nanocoatings from preclinical proof-of-concept toward certified clinical translation, future research must systematically address five key research avenues:

1. Research should prioritize lead-free, biodegradable, and mechanically compatible piezoelectric systems with enhanced polarization stability and high electromechanical conversion efficiency under weak physiological forces. Rational interface engineering, phase control, dipole alignment, nanofiller dispersion, and polarization-matched heterojunction construction will be essential to improve output while maintaining cytocompatibility.
2. Scalable and reproducible manufacturing methods are needed to support industrial-scale clinical translation. Fabrication techniques such as high-throughput electrospinning, electrophoretic deposition, micro/nano-patterning, 3D printing, plasma spraying, and magnetron sputtering should be optimized to achieve uniform coating thickness, stable crystal phase composition, consistent charge distribution, controllable surface morphology, and strong adhesion on complex, irregular implant surfaces.
3. Future studies must move beyond short-term monoculture antibacterial assays and adopt more clinically relevant testing platforms, including multispecies biofilms, dynamic fluid environments, cyclic mechanical loading systems, immune-cell co-culture platforms, and long-term large-animal infection models. These models are necessary to clarify how piezoelectric signals regulate host–pathogen interactions under realistic physiological conditions.
4. Standardized evaluation criteria should be established for piezoelectric anti-infective coatings, including piezoelectric output, charge stability, ROS production, antibacterial efficiency, antibiofilm performance, cytocompatibility, immunomodulatory effects, degradation behavior, and coating adhesion strength.
5. Regulatory and safety frameworks must define acceptable thresholds for in vivo microelectrical stimulation, reactive species generation, material degradation kinetics, and long-term toxicological risk.

### 5.3. Practical Clinical Implications and Clinical Outlook

From a clinical perspective, self-powered and wirelessly activated piezoelectric nanocoatings represent a versatile therapeutic platform with far-reaching practical applications. Piezoelectric coatings offer a non-invasive, site-specific therapeutic strategy adaptable to diverse clinical scenarios, including load-bearing orthopedic prostheses, dental implants, maxillofacial fixation hardware, vascular/urinary catheters, and active wound-healing matrices. In addition, by converting naturally occurring physiological motion (gait, joint loading, fluid flow) or non-invasive clinical excitation into localized antimicrobial action, these coatings physically disrupt recalcitrant biofilms and multidrug-resistant pathogens. This minimizes dependence on systemic antibiotics, lowers the risk of antimicrobial resistance, reduces implant revision surgery rates, and improves long-term prognosis for high-risk patients, such as diabetic or immunocompromised individuals. In the long term, combining piezoelectric nanocoatings with bio-sensing modules and wireless telemetry will enable next-generation theranostic implants. Such adaptive systems could continuously monitor localized microenvironmental cues (pH, temperature, bacterial metabolites) and trigger on-demand, self-regulated piezocatalytic therapy, establishing a new paradigm of personalized, site-specific infection control.

## Figures and Tables

**Table 1:** Representative piezoelectric antibacterial materials.

Material Properties	Piezoelectric Materials/Composites	Fabrication Method	Main Advantages	Key Engineering Challenges	References
Inorganic and composite materials	BaTiO_3_NPs	Co-precipitation/sol–gel/hydrothermal/solvothermal/high-energy milling	High piezoelectric coefficient; biocompatibility.	High brittleness; poor interfacial adhesion with metallic substrates	[35]
	Au@BTO NCs	Hydrothermal; precipitation			[36]
	BST-Ag	Hydrothermal; silver photodeposition	Antibacterial activity; enhanced osseointegration	Stimulation dependence	[38]
	BaCu_x_Ti_1−x_O_3_	Hydrothermal; copper doping			[39]
	BaTiO_3-x_/LA	Hydrothermal; defect engineering; silanization grafting			[40]
	SDBTO	Calcination			[41]
	BiFeO_3_-BaTiO_3_	Doping-defect engineering			[93]
	BaTiO_3_/HA	Powder blending; binder jetting 3D printing			[61]
	BaTiO_3_-SrZrTiO_3_	Sintering			[94]
	BT@PDA-La	Coating			[95]
	ZnO nanoarrays	Hydrothermal	Intrinsic antibacterial, piezoelectric, semiconducting properties	Dissolution	[48]
	BNNT-ZnO QDs	Sonication; solvothermal			[42]
	Ti-ZnO@CaP	Hydrothermal; Deposition			[49]
	ZnO@BT/HA	DLP 3D printing			[62]
	TaNTs@ZnO	Anodization; RF magnetron; deposition			[96]
	Na_0.5_K_0.5_NbO_3_ (NKN)	Solid-state sintering	Inertness and corrosion resistance.	Processing temperature Stability	[51]
	K_0.5_Na_0.5_NbO_3_ (KNN)	Solid-state reaction			[53]
	BG−xNKN	Sintering			[52]
	NaNbO_3_	Sol–gel dip-coating; sintering			[54]
	PEKK/BN@CO	Hydrothermal–calcination–composite–polarization			[55]
Organic and composite materials	PVDF-TrFE films	Flash annealing	Flexibility, lightweight, processability, and tissue-matched mechanics	Low piezoelectric coefficient, instability, and degradation.	[72]
	PVDF-TrFE PNFs	Electrospinning			[97]
	PVDF-g-PGAL	Alkali; oxidation; grafting			[71]
	TMCM-CdBrCl_2_/P(VDF-TrFE)	In situ growth; Electrospinning			[73]
	PVDF nanofibrous scaffolds	Electrospinning; drop-casting			[80]
	PVDF/TPU	Electrospinning			[76]
	PVDF/MXene	Electrospinning			[77]
	PVDF/RIVA	Electrospinning			[78]
	P(VDF-TrFE)/[Emim][HSO_4_]	Solvent casting			[98]
	PLLA	Electrospinning	Degradable and biocompatible	Low piezoelectric coefficient	[81,82]
	PLLA/ZA	Electrospinning			[83]
Organic–Inorganic composite materials	PLGA/Zn-KNN	Doping–composite–polarization	High output, flexible stability, biocompatibility	Aggregation, weak interfaces, poor reliability	[45]
	ZnO-composite	Deposition			[46]
	ZnO-PVDF-HFP	Dip-coating; annealing			[47]
	ZnO@PVDF/CS	Electrospinning			[79]
	PLLA/ZnO	Electrospinning			[88]
	KNN/PLLA	Electrospinning			[56]
	PVDF-TrFE/HAp	Electrospinning			[63]
	PLLA-HAp/PLLA	Electrospinning; doping			[64]
	Cu_3_B_2_O_6_/PVDF	Phase inversion			[74]
	PLLA/nHA	Electrospinning			[84]
	HA-KNN-CSL	Hydrothermal–blending–crosslinking			[86]
	PVDF/BaTiO_3_/CNT	Coating			[87]
	PLLA/CMBT	Electrospinning-casting			[89]
	P(VDF-TrFE)/ZnO/TPP	Hydrothermal; blade-coating			[90]
	E-MoS_2_/PVDF	Phase inversion			[91]
	PVA/PVDF/HA	Freeze–thaw cycling			[92]

**Table 2:** Antibacterial efficacy, stimulation conditions and immunomodulation of piezoelectric nanocoatings.

Piezoelectric Formulation	External Stimulus	Microorganism	Biological Classification	Primary Antibacterial/Antibiofilm Mechanism	Immunomodulatory Outcome	Ref.
TiO_2_/Bi_2_WO_6_ heterojunction arrays	NIR irradiation+ endogenous mechanical forces	*S. aureus*	Gram-positive bacterium	Separation through intrinsic electric field; ROS generation; antibacterial oxidative stress	Promotes osteogenic differentiation and implant osseointegration	[103]
Au@BTO nanocubes	Ultrasound-induced	*S. aureus*; *E. coli*	Gram-positive/Gram-negative bacterium	Schottky barrier; ROS regeneration	Reduces tissue inflammation	[34]
BTO-containing piezoelectric hydrogels	Mechanical deformation	*P. gingivalis* biofilm	Gram-negative bacterium	Transient surface charge modulation; inhibition of early bacterial adhesion	Enhanced bone marrow stem cell viability and osteogenic differentiation	[109]
piezoPCL film	Ultrasound stimulation	*S. aureus*	Gram-positive bacterium	NO-driven self-propulsion; enhanced biofilm penetration; piezoelectric ROS burst; EPS matrix disruption and persister eradication	/	[111]
BiOI/MXene piezoelectric catalytic bioheterojunction	Ultrasound stimulation	*S. aureus*; *E. coli*	Gram-positive/Gram-negative bacterium	Sonoluminescence-assisted charge separation; ROS; disruption of bacterial electron transport chain	First inducing M1 polarization, subsequently promoting M2 polarization	[112]
PLGA/BP/V_2_C bio-HJ nanocatalytic membrane	Mechanical/acoustic activation	*S. aureus*; *E. coli*; drug-resistant bacteria *Escherichia coli* strain	Gram-positive/Gram-negative bacterium	Suppression of bacterial complex IV activity; blocked respiratory electron flux; ATP starvation	Dampening the NF-κB inflammatory pathway	[113]
BTO-Ti	Low-intensity ultrasound	*P. gingivalis* biofilm	Gram-negative bacterium	Enhanced antibacterial activity; bioelectrical activation at implant–bacteria interface	Amplifies macrophage phagocytosis via PI3K-AKT and MAPK signaling	[115]
Advanced piezoelectric frameworks	Sustained low-level electrical stimulation after infection control	MRSA; *S. aureus*; *E. coli*; *C. albicans*	Gram-positive/Gram-negative bacterium	Support post-infectious antibacterial resolution and tissue repair	Promotes M2 polarization; up-regulates Arg-1, CD206, IL-4, and IL-10; enhances fatty acid oxidation and mitochondrial biogenesis	[108,117,^118^]
polyvinylidene fluoride/BaTiO_3_/MXene-Ti coating	Ultrasound drive	*S. aureus*; *E. coli*	Gram-positive/Gram-negative bacterium	Material-generated electrical signals and ROS assist pathogen clearance	Activates neutrophils through membrane potential modulation, Ca^2+^ influx, intracellular ROS burst, and NET formation	[120]
Pyroelectric functional coating on titanium implants, pyroTi	Daily intraoral temperature fluctuations; cold-induced thermal gradients			Bioelectrical stimulation through pyroelectric surface charge variation	Activates TRPM8 channels and Ca^2+^ influx in host stem cells; enhances early peri-implant bone remodeling	[119]
Resin-reinforced high-toughness piezoelectric BTO ceramic composite, SDIA	Daily occlusal loads	*S. aureus*; *E. coli*	Gram-positive/Gram-negative bacterium	Converts mechanical strain into stable bioelectric signals; ROS production	Upregulating the MAPK and PI3K-Akt signaling pathways	[121]
Ba/Ti-doped BiFeO_3_-BaTiO_3_ nanoreactor TH-BFBT	Acoustic drive	*S. aureus*; *E. coli*	Gram-positive/Gram-negative bacterium	ROS generation; accelerates Fe(III)/Fe(II) cycling; piezoacoustic-chemotherapeutic pathogen clearance	Supports bone scaffold anti-infection performance	[92]
TaNTs@ZnO	Physiological acidic infection microenvironment; piezoelectric ZnO response	*S. aureus*; *E. coli*	Gram-positive/Gram-negative bacterium	pH-responsive Zn^2+^ release; disrupts bacterial membranes and metabolic pathways	Preserves porous macro-topography for tissue integration	[95]
Zr single-atom-doped SrTiZrO_3_/hydroxyapatite heterojunction, STZO/Hap	Ultrasound stimulation	*S. aureus*; *E. coli*	Gram-positive/Gram-negative bacterium	ROS generation; Physical puncture; Metalloimmunotherapy effect	Released Zr^4+^ ions provide immunotherapeutic activity	[122]
PVDF-TrFE film with ionic liquid [Emim][HSO_4_]	Physical movement-induced piezoelectric stimulation	*S. aureus*; *E. coli* biofilm	Gram-positive/Gram-negative bacterium	Contact-killing via the ionic liquid; Disrupts bacterial membranes	Upregulation of Ca^2+^ channel gene expressions; osteogenic differentiation markers (RUNX2, BMP2) upon electromechanical stimulation	[97]
Copper-containing modified barium titanate coating, PH-CpBT	Ultrasound stimulation	*S. aureus*; *E. coli* biofilm	Gram-positive/Gram-negative bacterium	Cu^2+^/Cu^+^ cycling; ROS regeneration; bacterial membrane disruption	Promotes angiogenesis and biomineralization via Cu, Ca and P ions	[115]
ZnO-PVDF-HFP coating	External ultrasound; tunable acoustic parameters	*S. aureus*; *E. coli* biofilm	Gram-positive/Gram-negative bacterium	Generates localized surface potentials; inhibits biofilm formation	Reduces inflammatory cytokines such as IL-6 and TNF-α	[46]
Flexible PVDF-TrFE piezoelectric nanofiber film, PNF	External stimulation	*E. coli* biofilms	Gram-negative bacterium	Breaks EPS molecular bonds; Enhances antimicrobial penetration	Modulates electro-microenvironment around gastrointestinal wounds	[96]

## Data Availability

No new data were created or analyzed in this study. Data sharing is not applicable to this article.

## Abbreviations

The following abbreviations are used in this manuscript:

IAIs	Implant-associated infections
ROS	Reactive oxygen species
EPS	Extracellular polymeric substance
MDR	Multidrug resistant
PZT	Lead zirconate titanate
BTO	Barium titanate
LA	L-arginine
MRSA	Methicillin-resistant *Staphylococcus aureus*
hBMSCs	Human bone marrow mesenchymal stem cells
PLGA	Poly(lactic-co-glycolic acid)
KNN	Potassium-sodium niobate
BMSCs	Bone marrow mesenchymal stem cells
PEEK	Polyetheretherketone
PLLA	Poly(L-lactic acid)
LIPUS	Low-intensity pulsed ultrasound
HA	Hydroxyapatite
PVDF	Poly(vinylidene fluoride)
PVDF-TrFE	Poly(vinylidene fluoride-trifluoroethylene)
PHB	Polyhydroxybutyrate
PVDF:TPU	PVDF: thermoplastic polyurethane
NIR	Near-infrared
CS	Chitosan
PM	Particulate matter
ZA	Zolaphosphonic acid
PVA	Poly(vinyl alcohol)
US	Ultrasound
T-BTO	Tetragonal barium titanate
PDA	Polydopamine
ETC	Electron transport chain
PiG	Peri-implant gingiva
NETs	Neutrophil extracellular traps
CAUTIs	Catheter-associated urinary tract infections
